# Supplementary material for: Antibiotic use and survival from breast cancer: A population-based cohort study in England and Wales
Source: Nat Commun. 2025 Dec 21;16:11336. doi: 10.1038/s41467-025-67800-7 (PMC12722697; doi:10.1038/s41467-025-67800-7)
Supplement: Supplementary file 1 — Supplementary Information [file 41467_2025_67800_MOESM1_ESM.pdf]

Supplementary Online Content

CR Cardwell, S Baxter, AJHL Snelling, DT Chen, EC Atakpa, Ú McMenamin, SA McIntosh, B Hicks, CAC Coupland, AJ Brady, FJ Bannon, J Hippisley-Cox. Antibiotic use and survival from breast cancer: A population-based cohort study in England and Wales.

Supplementary Figure 1. Figure illustrating the study design for the analysis of antibiotic user versus non-user after diagnosis.

Supplementary Table 1. Additional sensitivity analyses for the association between antibiotics and breast cancer-specific mortality.

Supplementary Table 2. Additional subgroup analyses for the association between antibiotics and breast cancer-specific mortality.

STATA code for main analysis

Supplementary Figure 1. Figure illustrating the study design for the analysis of antibiotic user versus non-user after diagnosis.

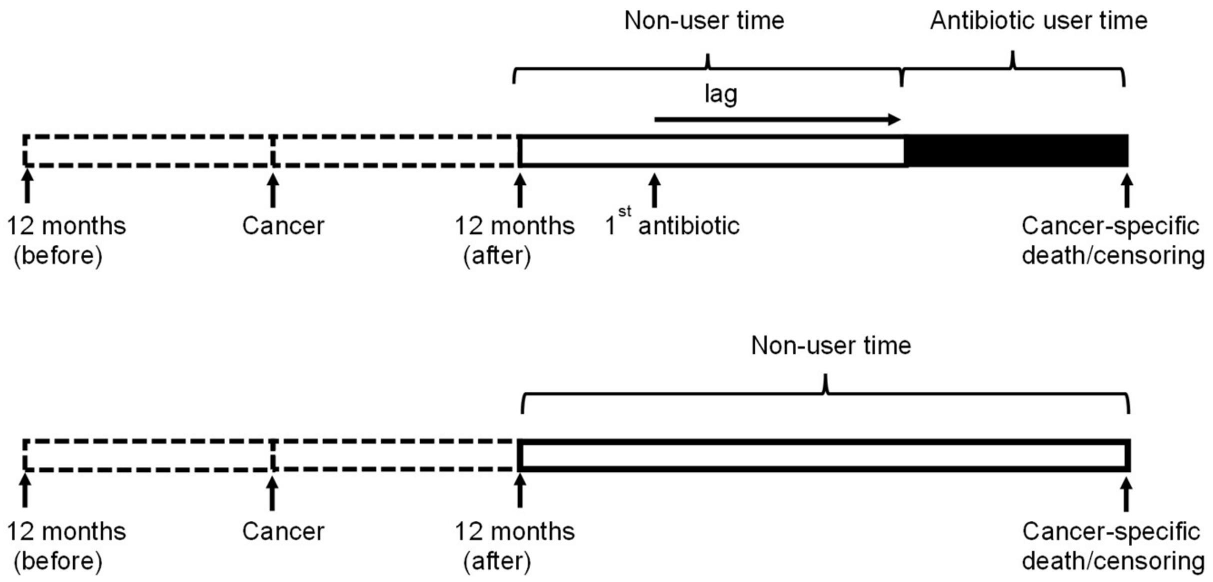

Supplementary Table 1. Additional sensitivity analyses for the association between antibiotics and breast cancer-specific mortality.

ABs	England Unadjusted HR (95% CI)	England Adjusted[1] HR (95% CI)	Wales Unadjusted HR (95% CI)	Wales Adjusted[1] HR (95% CI)	P for hetero[2]	Pooled adjusted[1] HR (95% CI)	P
Main analysis (events=4577, person-years=323556)							
Any	1.45 (1.33, 1.59)	1.19 (1.08, 1.31)	1.01 (0.91, 1.12)	0.96 (0.86, 1.07)	0.004	1.07 (0.87, 1.33)	0.521
1-5	1.38 (1.26, 1.52)	1.15 (1.05, 1.27)	0.95 (0.86, 1.06)	0.93 (0.83, 1.04)	0.005	1.04 (0.84, 1.28)	0.749
6-11	1.86 (1.60, 2.17)	1.39 (1.19, 1.62)	1.19 (1.01, 1.39)	1.05 (0.88, 1.25)	0.018	1.21 (0.92, 1.59)	0.173
12+	2.43 (2.00, 2.95)	1.81 (1.48, 2.20)	1.70 (1.41, 2.05)	1.45 (1.19, 1.78)	0.127	1.62 (1.31, 2.01)	<0.001
Ignoring antibiotic use in year before diagnosis (events=7484, person-years=503230)							
Any	1.47 (1.37, 1.59)	1.24 (1.15, 1.34)	1.08 (0.99, 1.17)	1.02 (0.93, 1.12)	0.002	1.13 (0.93, 1.36)	0.208
1-5	1.38 (1.27, 1.49)	1.18 (1.09, 1.28)	1.01 (0.93, 1.11)	0.98 (0.89, 1.08)	0.004	1.08 (0.90, 1.29)	0.402
6-11	1.81 (1.62, 2.02)	1.42 (1.27, 1.59)	1.21 (1.07, 1.37)	1.10 (0.97, 1.26)	0.004	1.26 (0.98, 1.61)	0.072
12+	2.14 (1.89, 2.42)	1.77 (1.56, 2.01)	1.49 (1.31, 1.69)	1.36 (1.18, 1.57)	0.007	1.55 (1.20, 2.01)	<0.001
Adjusting for smoking and BMI with complete case (events=3395, person-years=251392)							
Any	1.44 (1.30, 1.60)	1.18 (1.06, 1.32)	1.22 (1.07, 1.38)	1.11 (0.96, 1.27)	0.479	1.15 (1.06, 1.26)	0.001
1-5	1.38 (1.24, 1.54)	1.15 (1.03, 1.29)	1.17 (1.02, 1.33)	1.09 (0.95, 1.26)	0.580	1.13 (1.03, 1.23)	0.007
6-11	1.75 (1.47, 2.08)	1.29 (1.08, 1.54)	1.33 (1.09, 1.61)	1.10 (0.89, 1.36)	0.261	1.20 (1.03, 1.40)	0.019
12+	2.42 (1.95, 3.02)	1.84 (1.47, 2.30)	2.01 (1.61, 2.51)	1.60 (1.26, 2.04)	0.410	1.73 (1.47, 2.03)	<0.001
Three-year lag (events=3061, person-years=238720)							
Any	1.35 (1.21, 1.51)	1.14 (1.01, 1.28)	1.08 (0.95, 1.22)	1.06 (0.92, 1.21)	0.401	1.10 (1.01, 1.20)	0.030
1-5	1.32 (1.17, 1.48)	1.12 (1.00, 1.26)	1.04 (0.92, 1.19)	1.04 (0.90, 1.19)	0.390	1.08 (0.99, 1.19)	0.077
6-11	1.49 (1.22, 1.83)	1.17 (0.95, 1.44)	1.23 (1.01, 1.50)	1.14 (0.92, 1.40)	0.859	1.15 (0.99, 1.34)	0.064
12+	2.06 (1.59, 2.66)	1.66 (1.28, 2.16)	1.44 (1.12, 1.84)	1.32 (1.02, 1.72)	0.223	1.48 (1.19, 1.86)	<0.001
Excluding antibiotics in the first 6 months after diagnosis (events=4225, person-years=301583)							
Any	1.49 (1.36, 1.64)	1.22 (1.11, 1.35)	1.17 (1.05, 1.30)	1.11 (0.99, 1.24)	0.189	1.17 (1.06, 1.29)	0.002
1-5	1.42 (1.29, 1.57)	1.18 (1.07, 1.30)	1.11 (0.99, 1.24)	1.07 (0.95, 1.20)	0.193	1.13 (1.02, 1.25)	0.019
6-11	1.94 (1.65, 2.28)	1.42 (1.20, 1.68)	1.40 (1.18, 1.66)	1.28 (1.07, 1.54)	0.434	1.35 (1.20, 1.53)	<0.001
12+	2.44 (1.98, 3.00)	1.88 (1.53, 2.32)	1.83 (1.50, 2.24)	1.54 (1.24, 1.91)	0.188	1.70 (1.40, 2.07)	<0.001

[1]Adjusted model contains age, year of diagnosis, deprivation, stage, grade, surgery, radiotherapy, chemotherapy, tamoxifen use (in year after diagnosis), aromatase inhibitor use (in year after diagnosis), Charlson comorbidities (before diagnosis), home replacement therapy (before diagnosis) and statin, aspirin, and metformin use (after diagnosis) and is based upon a complete case analysis, except where otherwise stated. [2]P-value for heterogeneity comparing adjusted hazard ratio for England and Wales.

Supplementary Table 2. Additional subgroup analyses for the association between antibiotics and breast cancer-specific mortality.

ABs	England		Wales		P for hetero[2]	Pooled adjusted[1] HR (95% CI)	P
	Unadjusted HR (95% CI)	Adjusted[1] HR (95% CI)	Unadjusted HR (95% CI)	Adjusted[1] HR (95% CI)			
Progesterone receptor positive (events=729, person-years=76839)							
Any	1.34 (1.06, 1.69)	1.11 (0.87, 1.41)	1.26 (0.98, 1.62)	1.14 (0.87, 1.50)	0.870	1.12 (0.94, 1.34)	0.214
1-5	1.23 (0.96, 1.57)	1.04 (0.81, 1.34)	1.19 (0.92, 1.54)	1.12 (0.85, 1.48)	0.711	1.08 (0.89, 1.29)	0.436
6-11	2.12 (1.46, 3.08)	1.44 (0.98, 2.12)	1.56 (1.07, 2.25)	1.21 (0.81, 1.80)	0.546	1.32 (1.00, 1.75)	0.049
12+	2.32 (1.34, 4.02)	1.86 (1.07, 3.22)	1.89 (1.22, 2.94)	1.38 (0.86, 2.21)	0.422	1.56 (1.09, 2.24)	0.015
HER2 receptor positive (events=384, person-years=24890)							
Any	1.22 (0.87, 1.70)	1.08 (0.77, 1.53)	1.12 (0.80, 1.55)	1.02 (0.72, 1.44)	0.805	1.05 (0.82, 1.34)	0.700
1-5	1.13 (0.80, 1.60)	1.02 (0.72, 1.46)	1.15 (0.82, 1.61)	1.08 (0.76, 1.54)	0.826	1.05 (0.82, 1.35)	0.682
6-11	1.39 (0.77, 2.51)	1.11 (0.60, 2.05)	0.82 (0.47, 1.45)	0.67 (0.37, 1.19)	0.241	0.85 (0.52, 1.40)	0.520
12+	3.72 (1.94, 7.14)	3.03 (1.56, 5.91)	1.25 (0.63, 2.47)	0.77 (0.37, 1.58)	0.006	1.54 (0.40, 5.90)	0.532

[1]Adjusted model contains age, year of diagnosis, deprivation, stage, grade, surgery, radiotherapy, chemotherapy, tamoxifen use (in year after diagnosis), aromatase inhibitor use (in year after diagnosis), Charlson comorbidities (before diagnosis), home replacement therapy (before diagnosis) and statin, aspirin, and metformin use (after diagnosis) and is based upon a complete case analysis, except where otherwise stated. [2]P-value for heterogeneity comparing adjusted hazard ratio for England and Wales.

STATA code for main analysis

Analysis code

```
*Loading breast cancer cohort with all variables required
use "breast cancer main analysis cohort", clear

*Adding one-year lag period
replace timetodead =timetodead-1

*Declaring data as survival data with failures as breast cancer specific death
stset timetodead, fail(bcspec_dead==1) id(alf_pe)

*Creating time varying definition of antibiotic user vs non user (binary definition)
*Initially creating a variable containing time to first antibiotic in years
gen timetoantib=((fantibintime-bc_diag)/365.25)

*Splitting the observations at the time of first antibiotic
stsplit newtimeantib,at(0) after(time=timetoantib)

/*Creating a binary variable (antibyn) to identify antibiotic user (antibyn==1) observations or non-
antibiotic user times (antibyn==0)*/
gen antibyn=1 if _t0>=timetoantib & timetoantib !=.
replace antibyn=0 if antibyn==.

/*Creating time varying variables for aspirin, statins, metformin, tamoxifen and aromatase
inhibitors (`i'yn=1indicates medicine user for that observation and  `i'yn=0 indicates medicine non
user */
foreach i in asp stat metformin tamox arom {
	gen timeto`i'=((f`i'intime-bc_diag)/365.25)
	stsplit newtime`i',at(0) after(time=timeto`i')
	gen `i'yn=1 if _t0>=timeto`i' & timeto`i'~=.
	replace `i'yn=0 if `i'yn==.
	}

*unadjusted analysis
stcox i.antibyn

*adjusted analysis
 stcox i.antibyn age year i.stage i.grade surgery radio chemo i.tamoxyn i.aromyn mi chf pvd cvd
dementia cpd ulcer hep diab hemiplegia ckd i.hrtprior i.metforminyn i.statyn i.aspyn i.deprivation
```

Variable description

Variable	Description
alf_pe	Unique identifier
bc_diag	Date of breast cancer diagnosis
bcspec_dead	Breast cancer specific death (1= yes, 0= no)
fantibintime	Date of the first prescription for antibiotics after breast cancer diagnosis
timetodead	Length of follow-up in years
faspintime	Date of the first prescription for aspirin after breast cancer diagnosis
fstatintime	Date of the first prescription for statins after breast cancer diagnosis
fmetforminintime	Date of the first prescription for metformin after breast cancer diagnosis
ftamoxintime	Date of the first prescription for tamoxifen after breast cancer diagnosis

faromintime	Date of first prescription for aromatase inhibitor after breast cancer diagnosis
age	Age at first breast cancer diagnosis (in years)
year	Year of first breast cancer diagnosis
stage	Cancer stage at first breast cancer diagnosis
grade	Grade of tumour at first breast cancer diagnosis
surgery	Breast cancer surgery in the first year after diagnosis (yes or no variable)
radio	Radiotherapy in the first year after diagnosis (yes or no variable)
chemo	Chemotherapy in the first year after diagnosis (yes or no variable)
mi	Myocardial infarction before breast cancer diagnosis (yes or no variable)
chf	Heart failure before breast cancer diagnosis (yes or no variable)
pvd	Peripheral vascular disease before breast cancer diagnosis (yes or no variable)
cvd	Stroke before breast cancer diagnosis (yes or no variable)
dementia	Dementia before breast cancer diagnosis (yes or no variable)
cpd	Chronic obstructive pulmonary disease before breast cancer diagnosis (yes or no variable)
ulcer	Peptic ulcer before breast cancer diagnosis (yes or no variable)
hep	Liver disease before breast cancer diagnosis (yes or no variable)
diab	Diabetes before breast cancer diagnosis (yes or no variable)
hemiplegia	Hemiplegia before breast cancer diagnosis (yes or no variable)
ckd	Chronic kidney disease before breast cancer diagnosis (yes or no variable)
hrtprior	Hormone replacement therapy before breast cancer therapy
deprivation	2011 Index of Multiple Deprivation in Wales and the 2011 Townsend deprivation score in England based on residential address at breast cancer diagnosis in quintiles

Further STATA code available from authors upon request.
