# Supplementary material for: Antibiotic use and survival from breast cancer: A population-based cohort study in England and Wales
Source: Nat Commun. 2025 Dec 21;16:11336. doi: 10.1038/s41467-025-67800-7 (PMC12722697; doi:10.1038/s41467-025-67800-7)
Supplement: Supplementary file 2 — Reporting Summary [file 41467_2025_67800_MOESM2_ESM.pdf]

nature portfolio

Corresponding author(s): Chris R Cardwell

Last updated by author(s): Nov 17, 2025

Reporting Summary

Nature Portfolio wishes to improve the reproducibility of the work that we publish. This form provides structure for consistency and transparency in reporting. For further information on Nature Portfolio policies, see our Editorial Policies and the Editorial Policy Checklist.

Statistics

For all statistical analyses, confirm that the following items are present in the figure legend, table legend, main text, or Methods section.

n/a	Confirmed	
☐	☒	The exact sample size (*n*) for each experimental group/condition, given as a discrete number and unit of measurement
☐	☒	A statement on whether measurements were taken from distinct samples or whether the same sample was measured repeatedly
☐	☒	The statistical test(s) used AND whether they are one- or two-sided<br>*Only common tests should be described solely by name; describe more complex techniques in the Methods section.*
☐	☒	A description of all covariates tested
☐	☒	A description of any assumptions or corrections, such as tests of normality and adjustment for multiple comparisons
☐	☒	A full description of the statistical parameters including central tendency (e.g. means) or other basic estimates (e.g. regression coefficient) AND variation (e.g. standard deviation) or associated estimates of uncertainty (e.g. confidence intervals)
☐	☒	For null hypothesis testing, the test statistic (e.g. *F*, *t*, *r*) with confidence intervals, effect sizes, degrees of freedom and *P* value noted<br>*Give P values as exact values whenever suitable.*
☒	☐	For Bayesian analysis, information on the choice of priors and Markov chain Monte Carlo settings
☒	☐	For hierarchical and complex designs, identification of the appropriate level for tests and full reporting of outcomes
☒	☐	Estimates of effect sizes (e.g. Cohen's *d*, Pearson's *r*), indicating how they were calculated

Our web collection on statistics for biologists contains articles on many of the points above.

Software and code

Policy information about availability of computer code

Data collection	Software was not used to collect the data. As described in the methods data were used from SAIL Databank and QResearch.
Data analysis	STATA version 18 was used to conduct analysis. STATA code for main analysis shown in Supplementary Material.

For manuscripts utilizing custom algorithms or software that are central to the research but not yet described in published literature, software must be made available to editors and reviewers. We strongly encourage code deposition in a community repository (e.g. GitHub). See the Nature Portfolio guidelines for submitting code & software for further information.

Data

Policy information about availability of data

All manuscripts must include a data availability statement. This statement should provide the following information, where applicable:

- Accession codes, unique identifiers, or web links for publicly available datasets
- A description of any restrictions on data availability
- For clinical datasets or third party data, please ensure that the statement adheres to our policy

The datasets from Wales (SAIL databank; Swansea University, https://saildatabank.com/) and England (QResearch; Queen Mary University of London, https://www.qresearch.org/) were based upon anonymised patient records and were obtained under strict data access conditions that allowed the study to be conducted but do not allow direct data sharing. Access to both SAIL databank and QResearch involve a fee and are subject to specific governance and licensing conditions. Full

details on how to request access, including contact information and procedures, are available at SAIL databank (Swansea University, https://saildatabank.com/) and QResearch (Queen Mary University of London, https://www.qresearch.org/).

Research involving human participants, their data, or biological material

Policy information about studies with human participants or human data. See also policy information about sex, gender (identity/presentation), and sexual orientation and race, ethnicity and racism.

Reporting on sex and gender	The study investigated women only.
Reporting on race, ethnicity, or other socially relevant groupings	In the study deprivation was a covariate.
Population characteristics	The study investigates women over the age of 18, who were diagnosed with breast cancer between 2000 and 2017 in England and Wales.
Recruitment	The study is based upon routinely collected data.
Ethics oversight	Ethical approval for the QResearch database is obtained annually from East Midlands - Derby Research Ethics Committee (Ref: 18/EM/0400). Approval for our analysis of the English data has been obtained from the QResearch Scientific Committee. Approval for analysis of the Welsh data has been obtained from the SAIL Databank Information Governance Review Panel.

Note that full information on the approval of the study protocol must also be provided in the manuscript.

Field-specific reporting

Please select the one below that is the best fit for your research. If you are not sure, read the appropriate sections before making your selection.

☒ Life sciences ☐ Behavioural & social sciences ☐ Ecological, evolutionary & environmental sciences

For a reference copy of the document with all sections, see nature.com/documents/nr-reporting-summary-flat.pdf

Life sciences study design

All studies must disclose on these points even when the disclosure is negative.

Sample size	In advance we estimated the Welsh breast cancer cohort would contain over 17,000 stage 1 to 3 breast cancer patients newly diagnosed from 2000 to 2017 in whom there would be 2,125 cancer-specific deaths. In England we estimated there would be over 60,000 stage 1 to 3 breast cancer patients in whom we would expect 7,500 cancer-specific deaths, based upon Welsh cancer-specific mortality rates. Antibiotic prescribing prevalence was estimated from a previous case control study. In the year before breast cancer diagnosis an estimated 30% of breast cancer patients received an antibiotic prescription, in a 5 year period 55% of all breast cancer patients received an antibiotic prescription and 4% received 12 or more antibiotic prescriptions.<br><br>Using Schoenfeld's method, based upon the numbers above, we would have over 95% power to detect a, clinically important, relative 20% increase (i.e. a HR of 1.2) in breast cancer-specific mortality in antibiotic users compared with non-users after diagnosis. Similarly, in users of 12 or more antibiotics we could detect a 25% increase in cancer-specific mortality and by antibiotic class we could detect a 20% increase in cancer-specific mortality with use of cephalosporins (the antibiotic type used in the mouse model experiments) or penicillin (based upon 10% use of cephalosporins and 33% use of penicillin). See methods for more details.
Data exclusions	Patients previously diagnosed with other invasive cancer diagnoses (apart from non-melanoma skin cancer) were excluded. Patients who were not registered at a GP on the date of their breast cancer diagnosis or who had less than a year of GP records prior to the date of their breast cancer diagnosis were excluded.
Replication	The research was conducted separately in Wales and England and similar results were observed. See results section.
Randomization	It was not possible to use randomization in this observational study.
Blinding	It was not possible to use blinding in this observational study.

Reporting for specific materials, systems and methods

We require information from authors about some types of materials, experimental systems and methods used in many studies. Here, indicate whether each material, system or method listed is relevant to your study. If you are not sure if a list item applies to your research, read the appropriate section before selecting a response.

Materials & experimental systems

n/a	Involved in the study
☒	☐ Antibodies
☒	☐ Eukaryotic cell lines
☒	☐ Palaeontology and archaeology
☒	☐ Animals and other organisms
☒	☐ Clinical data
☒	☐ Dual use research of concern
☒	☐ Plants

Methods

n/a	Involved in the study
☒	☐ ChIP-seq
☒	☐ Flow cytometry
☒	☐ MRI-based neuroimaging

Plants

Seed stocks

Report on the source of all seed stocks or other plant material used. If applicable, state the seed stock centre and catalogue number. If plant specimens were collected from the field, describe the collection location, date and sampling procedures.

Novel plant genotypes

Describe the methods by which all novel plant genotypes were produced. This includes those generated by transgenic approaches, gene editing, chemical/radiation-based mutagenesis and hybridization. For transgenic lines, describe the transformation method, the number of independent lines analyzed and the generation upon which experiments were performed. For gene-edited lines, describe the editor used, the endogenous sequence targeted for editing, the targeting guide RNA sequence (if applicable) and how the editor was applied.

Authentication

Describe any authentication procedures for each seed stock used or novel genotype generated. Describe any experiments used to assess the effect of a mutation and, where applicable, how potential secondary effects (e.g. second site T-DNA insertions, mosiacism, off-target gene editing) were examined.
